# Extrusion-Printing of Multi-Channeled Two-Component Hydrogel Constructs from Gelatinous Peptides and Anhydride-Containing Oligomers

**DOI:** 10.3390/biomedicines9040370

**Published:** 2021-04-01

**Authors:** Jan Krieghoff, Johannes Rost, Caroline Kohn-Polster, Benno M. Müller, Andreas Koenig, Tobias Flath, Michaela Schulz-Siegmund, Fritz-Peter Schulze, Michael C. Hacker

**Affiliations:** 1 Institute of Pharmacy, Pharmaceutical Technology, Faculty of Medicine, University of Leipzig, Eilenburger Straße 15a, 04317 Leipzig, Germany; jan.krieghoff@uni-leipzig.de (J.K.); schulz@uni-leipzig.de (M.S.-S.); 2 Department of Mechanical and Energy Engineering, Leipzig University of Applied Sciences (HTWK Leipzig), Karl-Liebknecht-Straße 134, 04277 Leipzig, Germany; tobias.flath@htwk-leipzig.de (T.F.); peter.schulze@htwk-leipzig.de (F.-P.S.); 3 Department of Prosthodontics and Materials Science, University of Leipzig, Liebigstraße 12, 04103 Leipzig, Germany; Andreas.Koenig@medizin.uni-leipzig.de; 4 Institute of Pharmaceutics and Biopharmaceutics, Heinrich Heine University, Universitätsstraße 1, 40225 Düsseldorf, Germany

**Keywords:** multi-channeled nerve guidance conduit, additive manufacturing, two-component hydrogel, reactive oligomer, in vitro degradation

## Abstract

The performance of artificial nerve guidance conduits (NGC) in peripheral nerve regeneration can be improved by providing structures with multiple small channels instead of a single wide lumen. 3D-printing is a strategy to access such multi-channeled structures in a defined and reproducible way. This study explores extrusion-based 3D-printing of two-component hydrogels from a single cartridge printhead into multi-channeled structures under aseptic conditions. The gels are based on a platform of synthetic, anhydride-containing oligomers for cross-linking of gelatinous peptides. Stable constructs with continuous small channels and a variety of footprints and sizes were successfully generated from formulations containing either an organic or inorganic gelation base. The adjustability of the system was investigated by varying the cross-linking oligomer and substituting the gelation bases controlling the cross-linking kinetics. Formulations with organic *N*-methyl-piperidin-3-ol and inorganic K_2_HPO_4_ yielded hydrogels with comparable properties after manual processing and extrusion-based 3D-printing. The slower reaction kinetics of formulations with K_2_HPO_4_ can be beneficial for extending the time frame for printing. The two-component hydrogels displayed both slow hydrolytic and activity-dependent enzymatic degradability. Together with satisfying in vitro cell proliferation data, these results indicate the suitability of our cross-linked hydrogels as multi-channeled NGC for enhanced peripheral nerve regeneration.

## 1. Introduction

Peripheral nerve regeneration is one of the fields of regenerative medicine in which structural cues on micro- and macroscopic levels were used to improve clinical outcomes during the last years [1]. For nerve guidance conduits (NGC) that have been composed of natural and synthetic materials, it has been shown that the guidance ability and biochemical communication between the proximal and distal ends of neurons are currently only satisfying in small-scale defects (<10 mm) [2]. As a strategy to overcome such limitations, the implementation of an array of smaller channels within the NGC lumen has been promoted [3]. Such multi-channeled conduits imitate the nerve architecture as they limit the space into which the regenerating axons could grow by their structure, prevent dispersion of biochemical signal molecules and offer topographical guidance cues [4]. The fabrication of such conduits is more complex, and a broad variety of techniques has been investigated [5]. Early approaches used stainless steel wires in molds [6,7], while more recent approaches fabricated multi-channeled conduits via stereolithographic printing [8] or controlled freeze-drying [9,10].

Fabrication of three-dimensional (3D) structures via additive manufacturing techniques such as 3D-printing allows for the generation of structures with complex internal geometries and enables flexible control over the dimensions and even material composition of a construct [11]. The generic term 3D-printing encompasses a broad variety of techniques and working principles that can be adapted to a wide range of materials and material composites including hydrogels [12,13,14]. Most commercial bioprinters are equipped with a printhead for extrusion printing of viscous liquids by a motor-driven plunger or pressurized air [15]. Extrusion-based printing is a low complexity approach that requires a material that is stable enough to hold the desired shape after extrusion while remaining printable and reactive for the covalent fusion of subsequently extruded layers [16,17,18]. These different levels of solidification can be realized in different approaches, for example by multi-step cross-linking of a hydrogel based on photo-cross-linkable hyaluronic acid and alginate that is extruded into a viscous suspension medium containing calcium ions for ionic alginate gelation and subsequent UV-treatment for photo-cross-linking [19,20]. By incorporation of a phosphate ion source into the printing mixture, it is even possible to in situ generate CaP nanoparticles, yielding a nanocomposite hydrogel with improved mechanical stability [19]. Another approach incorporates inorganic laponite nanoclay as a filler into a gelatin/oxidized alginate hydrogel to stabilize printed structures following shear thinning during printing [21].

For the generation of multi-channel NGC by extrusion-based printing gelatin peptides are appealing as they are biodegradable, exhibit low immunogenicity and cytotoxicity and possess motifs that promote interaction with cells [22,23]. Gelatin-type peptides are naturally capable of forming physical hydrogels [24]. However chemical cross-linking of gelatin chains is used to strongly enhance and tune the resulting hydrogel stability (e.g., mechanical, hydrolytic) [25]. Short gelatin-like peptides like Collagel^®^ (GEL) do not show a temperature-dependent sol–gel transition and require chemical cross-linking for hydrogel formation [26]. Common chemical cross-linkers include 1-ethyl-3-(3-dimethylaminopropyl) carbodiimide and *N*-hydroxy-succinimide [27], glutaraldehyde [28] and genipin [29]. Depending on the mass ratio of cross-linker to polypeptides, cross-linkers that are integrated into the network can significantly alter the properties of the final hydrogel [23]. Hence, it is more suitable to consider a hydrogel with a high cross-linker content as a two-component hydrogel.

Loth et al. developed synthetic oligomers that contain anhydride functionalities for the cross-linking of amine-containing macromolecules like gelatinous peptides [30]. Residual anhydrides are eventually hydrolyzed in an aqueous environment, rendering non-reacted cross-linking sites inert. These oligomers enabled the generation of stable two-component hydrogels with theoretical mass ratios of oligomer to gelatinous peptides ranging from 1:5 up to 1:1.36 while properties were dependent on both building blocks [26]. The cross-linking reaction between the primary gelatinous amines and anhydrides of the oligomer requires a base to scavenge the protons of the concurrently liberated carboxyl functionalities and prevent protonation of remaining amines. Previously, organic bases like triethylamine (TEA) [26,31] and *N*-methyl-piperidin-3-ol (NMPO) [32] were used for this purpose. In general, oligomers with a high anhydride content can be partially pre-derivatized with amine-containing small molecules to alter the properties of the resulting hydrogels, for instance with cationic moieties to improve cellular attachment [32,33]. In addition to the initial oligomers containing *N*-isopropylacrylamide (NiPAAm) as co-monomer (oPNMA), a second type of oligomer with the carbonyl functionality-containing diacetone acrylamide (DAAm) as co-monomer (oPDMA) was developed to enable further postfabrication functionalization [31].

The main aim of this work was to investigate extrusion-based 3D-printing from a single cartridge of a two-component hydrogel for the generation of multi-channeled structures. As no complex printhead for in-process mixing was required to process our full-gelation-premixture, this approach is feasibly adaptable to most commercial bioprinters with an extrusion printhead. The full-gelation-premixture was obtained by mixing a solution of the gelatinous peptides with base with a solution of the oligomer. The printing process started once a stable strand was extruded while ongoing anhydride-amine conversion of the full-gelation-premixture enabled cross-linking and adherence between deposited strands during continuous printing. Different printing patterns were established that allowed for extrusion of multi-channeled structures with rectangular or circular cross-sections. The resulting two-component hydrogel constructs were characterized regarding mass increase per layer, volume and channel structure. Pristine and pre-derivatized oligomers as well as inorganic phosphates were used to control the cross-linking reaction and expand the adaptability of the system. In addition to 3D-printing, the inorganic phosphates were also studied in detail with conventionally fabricated hydrogels. In vitro degradation experiments in absence and presence of a collagen-degrading enzyme were performed to explore the degradability of the cross-linked two-component hydrogels. Finally, the ability to aseptically 3D-print two-component hydrogel constructs and the cytocompatibility of aseptically printed constructs were investigated.

## 2. Materials and Methods

### 2.1. Synthesis and Characterization of the Anhydride-Containing Oligomer

The synthesis and characterization of anhydride-containing oligomers oligo(PEDAS-*co*-MA-*co*-NiPAAm) (oPNMA) and oligo(PEDAS-*co*-MA-*co*-DAAm) (oPDMA) was performed as previously described (Supplementary Figure S1 and Supplementary Table S1) [30,31].

In the oligomer code oPxMA-z, z refers to the feed of maleic anhydride (MA) in the 20 eq of MA and respective co-monomer. For instance, oPNMA-10 is synthesized from a monomer mix of 1 eq of pentaerythritol diacrylate monostearate (PEDAS)), 10 eq of MA and 10 eq of NiPAAm.

### 2.2. Manual Fabrication of Two-Component Hydrogels

Two slightly different methods were employed for the manual preparation of oligomer-cross-linked two-component hydrogels. Both methods used the same components: a GEL solution in water, an oligomer solution in *N*-dimethylformamide (DMF) and an organic or inorganic base diluted to the required concentration in water or DMF. The reaction vessels, flat-bottom glass vials, were identical in both methods.

*Two-component hydrogel fabrication by direct mixing:* As previously described [26,32], 200 µL cross-linker solution were pipetted to 50 µL base solution in the reaction vessel. GEL solution (200 µL) was added, and the vessel was shaken with a vortex mixer for 5 s.

*Two-component hydrogel fabrication from a GEL-base premix*: Four parts of 1.25-fold concentrated GEL solution were first thoroughly mixed with 1 part concentrated base solution (5-fold). Equal amounts (200 µL each) of the resulting premix and oligomer solution were added to the reaction vessel and shaken for 5 s.

For both methods, the fabricated two-component gels were left to dry for two days at ambient conditions and one day at reduced pressure (600 mbar).

### 2.3. 3D-Printing of Two-Component Hydrogels

Extrusion-based 3D-printing of the two-component hydrogels was performed using a BioScaffolder^®^ (SYSENG, Salzgitter-Bad, Germany) with a single cartridge stepper motor driven printhead (type 4032, axis GmbH, Keltern, Germany). In this printhead, a 3 mL Luer-Lock-syringe (CODAN Medizinische Geräte GmbH & Co KG, Lensahn, Germany) with a Micron-S dispensing tip (0.16 mm inner diameter, VIEWEG GmbH, Kranzberg, Germany) was mounted. Patterns with both a rectangular and a circular footprint were printed. To ensure extrusion of a homogenous hydrogel strand, a short linear pattern was extruded prior to construction of the first structure.

For the preparation of the two-component hydrogel full-gelation-premixture, 9 parts of 1.11-fold concentrated GEL solution in water (typically 900 µL of 33.3% (w/V)) were thoroughly mixed with 1 part 10-fold concentrated base solution in water (typically 100 µL of 1.04 M). A timer was started with the addition of 10 parts oligomer solution in DMF (typically 1000 µL of 7% (*w/v*)), and the full-gelation-premixture was immediately homogenized on a shaker. After complete homogenization, the full-gelation-premixture was aspirated into the syringe, which was then placed into the printhead. Construct extrusion was started after the preprint time window of 12 min had passed. The full-gelation-premixture was dispensed at a rate of 0.5 µL/s, and the printhead movement rate was 6 mm/s for rectangular outlines and 8 mm/s for circular outlines. For the typical composition, the printing window was at least 70 min after start of printing, enough to use up an enlarged full-gelation-premixture of 1400 µL of each component.

Printed constructs were measured, weighed and left to dry for 2 days at ambient conditions and 1 day at reduced pressure (600 mbar).

### 2.4. Post-Fabrication Processing and Characterization of Two-Component Hydrogels

#### 2.4.1. Washing and Lyophilization of Dried Hydrogels

Both manually fabricated and 3D-printed two-component hydrogels were hydrated in Dulbecco’s phosphate buffered saline (PBS) (preserved with 0.1 g/L sodium azide when handling hydrogels under non-sterile conditions) after drying. The buffer was replaced after 0.5, 1 and 2 days to remove non-cross-linked components and bases. Following final buffer removal after 3 days total, hydrogels were frozen at −20 °C prior to lyophilization (Christ Alpha 2-4 lyophilizer, Martin Christ Gefriertrocknungsanlagen GmbH, Osterode am Harz, Germany).

#### 2.4.2. Hydrogel Weight, Water Content and Leachables

Characterization of manually fabricated hydrogels was performed as previously described [26]. Complete hydrogels were weighed after lyophilization (m_dry_) and subsequently rehydrated using conserved PBS. Disks with 8 mm diameter were punched out and weighed following removal of excess buffer (m_wet disk_). Afterwards, disks were frozen, lyophilized and weighed again in the dry state (m_dry disk_).

All printed hydrogels were weighed following final buffer removal after carefully blotting off excess buffer (m_wet,printed_) and after lyophilization (m_dry,printed_). Furthermore, the dimensions of the constructs were measured at these instances.

Water contents, salt-corrected dry masses and, for manually fabricated hydrogels, the contents of water leachable components were calculated using corresponding wet and dry masses.

#### 2.4.3. Rheological Characterization of Fabricated Hydrogels

The 8 mm disks were employed to characterize the mechanical properties of the two-component hydrogels by oscillation rheology as previously described [26]. Experiments were performed with an 8 mm steel plate geometry (Physica MCR 301 rheometer, Anton Paar GmbH, Graz, Austria) with disks swollen to the equilibrium state in conserved PBS. Discs were placed in the instrument and the geometry lowered until a normal force of 0.2 N was reached. Subsequently, a frequency sweep from 0.1 to 10 Hz was performed at 37 °C with a constant strain of 1% at which the gels were expected to display linear viscoelastic behavior. Storage modulus (G’), loss modulus (G’’) and complex viscosity (η*) were recorded.

#### 2.4.4. Stereomicroscopic Visualization

Detailed visualization of fabricated constructs was done using a SM33 stereomicroscope (Hund Wetzlar, Wetzlar, Germany). Pictures were documented using DS-2Mv camera and the NHS-Elements software (version 4.6, Nikon, Duesseldorf, Germany).

### 2.5. Kinetics of the Cross-Linking Reaction

Oscillation rheology was further employed to characterize the kinetics of the cross-linking reaction of the two-component hydrogels as previously described [32]. Experiments were performed with 25 mm diameter cone geometry (CP25-1, 1° cone angle, Anton Paar GmbH, Graz, Austria) with a gap size of 0.048 mm at 20 °C. A solution (50 µL) of 4 parts 1.25-fold concentrated GEL solution and 1 part 5-fold concentrated base was applied to the bottom plate of the assembly. Then, 50 µL cross-linker solution were added centrally. Storage modulus (G’), loss modulus (G’’) and complex viscosity (η*) at 1 Hz frequency and an amplitude of 1% were recorded every 30 s for 15 min following a 10 s mixing step.

### 2.6. Pre-Derivatization of Anhydride-Containing Oligomer

For pre-hydrogel fabrication pre-derivatization of a part of the anhydrides of the oligomers, oPNMA-10 with 10 equivalents (eq.) of intact MA was dissolved to 105 mg per mL in DMF (typical intact MA concentration 193 µmol/mL) [32]. *N,N*-diethylethylenediamine (DEED) was diluted in DMF to a final concentration of 7.5 eq. per mL (typical concentration: 96.5 µmol/mL). Two parts cross-linker solution and 1 part DEED solution were combined to yield 2.5 eq. DEED per 10 eq. intact anhydrides. After incubation at ambient temperature for 30 min, pre-derivatized oligomer was employed for hydrogel fabrication as described above.

### 2.7. Degradation Analysis

Fabricated two-component hydrogel constructs were investigated for their in vitro degradation behavior. Dry constructs were weighed, dimensionally characterized and placed in pre-weighed glass vials.

*Hydrolytic degradation*: Five milliliters of preserved PBS (pH 7.4) was added to each vial, resulting in a ratio of sample weight (in mg) to buffer volume (in µL) of about 1:125-167. Initially, vials were kept at ambient temperature for 2 h for rehydration, after which degradative behavior was observed at 37 °C. Buffer was replaced with 5 mL of fresh PBS every week.

*Enzyme-mediated degradation*: The same set-up was employed for the investigation of enzyme-mediated degradation. Preserved PBS that was augmented with 0.1 mM CaCl_2_ and MgCl_2_ and defined activities of Collagenase A from *Clostridium histolyticum* served as degradation medium. Initially, constructs were rehydrated in non-enzyme-containing buffer at ambient temperature for 6 h. Subsequently, the buffer was exchanged for enzyme-containing buffer, and degradation was examined at 37 °C. Visual changes were documented every day.

For both degradation conditions, samples were taken at defined time points, weighed (wet weight), frozen, lyophilized and weighed again (dry weight). The wet and dry masses were used to calculate salt-corrected dry masses as described above.

### 2.8. µXCT Analysis of Degradation Samples

Micro X-ray computer tomography (µXCT) was used to visualize the microstructure in three dimensions and to quantify the total porosity. The μXCT (FhG, Dresden, Germany) was equipped with an X-ray tube FXE 225.99 (focal spot diameter 0.6 µm, tungsten target) by YXLON International GmbH (Hamburg, Germany) and a 2D-detector 1621xN (2048 × 2048 pitches, CsI, pitch size 200^2^ µm^2^) by PerkinElmer (PerkinElmer LAS (Germany) GmbH, Rodgau, Germany). The samples were measured with an X-ray power of 4.4 Watt (beam energy 160 kV and flux 150 μA), with no filter and a step size of 0.45/360 (800 positions). The voxel edge length as an indicator for maximum resolution was maintained at 12 μm (*V* = 1728 μm^3^).

The grey-value-specific three-dimensional raw data sets were cut and orientated with ImageJ (version 1.47, National Institutes of Health). The segmentations between material and background were realized with the Otsu algorithms [34]. Taking into account the resolution, the total porosity of cross sections (6.5^2^ mm^2^, 400 sections) was determined for every sample based on the calculated thresholds.

### 2.9. Fabrication under Aseptic Conditions and Analysis for Microbiological Contamination

Aseptic fabrication was achieved in a laminar air flow workbench. The setup was thoroughly cleaned and disinfected. The solutions of oligomer, GEL and base were filtered through 0.22 µm polyethersulfone filters prior to fabrication. Post-fabrication processing was performed under aseptic conditions with sterile PBS. Following lyophilization, selected samples were additionally γ-sterilized with a dose of 15 kGy (BBF Sterilisationsservice GmbH, Kernen-Rommelshausen, Germany).

Sterility testing according to the European Pharmacopeia 9.0 (Ph. Eur.), 2.6.1 for aerobic bacteria and fungi, was performed in an external certified laboratory (SYNLAB Analytics & Services Germany GmbH, Leipzig-Markkleeberg, Germany). Samples were immersed in casein soybean digest broth for 14 days at 20–25 °C. During and after incubation, microbial growth was assessed. *Aspergillus brasiliensis*, *Bacillus subtilis* and *Candida albicans* were employed as positive controls.

### 2.10. Direct Contact Cell Culture of hASC on Two-Component Hydrogels

The direct in vitro cell culture of primary human adipose-derived stem cells (hASC) on the two-component hydrogels were investigated as previously published [33]. Experiments were performed on manually fabricated 11 mm hydrogel discs that were rehydrated in cell culture medium with 1% (*v/v*) penicillin/streptomycin (P/S) as supplement at 37 °C for 24 h. Silicone O-rings were used to keep gels discs submerged in medium in the cavities of 48 well plates.

For hASC culture, 10^4^ cells (passage 5–7) in 1 mL Dulbecco’s Modified Eagle Medium (DMEM) low glucose enriched with 10% (*v/v*) fetal bovine serum (FBS) and 1% (*v/v*) P/S were seeded per hydrogel disc in a standard 48 well plate and incubated at 37 °C in a 5% CO_2_ atmosphere. Following 3 days of cell cultivation with daily medium exchange, cells were washed with PBS, fixated with 3.7% (*v/v*) paraformaldehyde and permeabilized with 0.5% (*v/v*) Triton X-100. Cells were stained with DAPI (300 nM, 1:1000) and Alexa Fluor^®^ 488 phalloidin (1:40) according to the manufacturer’s protocol and examined by fluorescence microscopy (LSM 700, Zeiss, Germany).

### 2.11. Indirect Cytocompatibility Testing

Indirect quantification of the cytocompatibility of aseptically fabricated constructs was performed with L929 mouse fibroblasts according to ISO 10993-5 and the previously published protocol [32]. Constructs (*n* = 5) were rehydrated in 7 mL DMEM low glucose without phenol red per construct at 37 °C in a humidified incubator for 24 h. The medium was discarded, and 1 mL fresh medium was added to remove excess PBS salt residues from the washing process. After 24 h incubation, the conditioned medium was removed and filtered (polyethersulfone, 0.2 µm pore size). L929 fibroblasts (passage 38) were seeded in a 96-well plate at 10^4^ cells/well. Cells were cultivated in DMEM low glucose with 10% (*v/v*) FBS and 1% (*v/v*) P/S in a 5% CO_2_ atmosphere at 37 °C for 24 h. Subsequently, medium was exchanged for conditioned or fresh culture medium. After 24 h incubation, the metabolic activity was quantified by Alamar Blue^®^ assay as published [32]. Afterwards, fresh culture medium was added to all wells, and the metabolic activity was reassessed after 48 h. The cell viability was calculated by relating the fluorescence intensity of the test groups to the positive controls (each *n* = 5).

### 2.12. Statistical Analysis

All statistical analysis was performed using GraphPad Prism 8.0 software (San Diego, CA, USA). Results are given as mean ± standard deviation (SD). Analysis of variance (ANOVA) tests assuming no equal standard deviations (Brown-Forsythe and Welch) were used to evaluate differences. Null Hypothesis for statistical tests assumed that there is no difference between the analyzed values, and 95% was assumed to be the significance level.

## 3. Results and Discussion

### 3.1. Two-Component Hydrogels with Inorganic Bases

**Figure 1:**
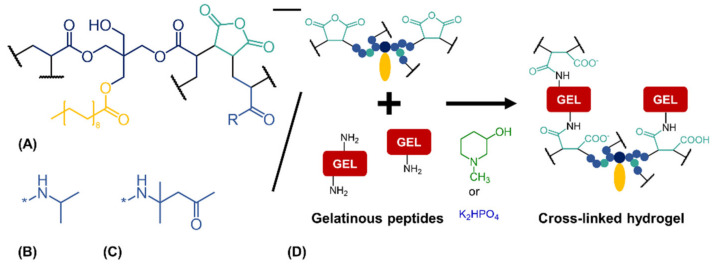
Cross-linking reaction between anhydride-containing oligomers and gelatinous peptides. (**A**) Cross-linking oligomer with co-monomers (**B**) *N*-isopropyl residue (R) as in oPNMA or (**C**) residue (R) of diacetone acrylamide as used in oPDMA. (**D**) Base-catalyzed reaction between anhydrides of the oligomers and amines of the peptides to yield cross-linked two-component hydrogels.

The basis for the fabrication of a stable two-component hydrogel is the cross-linking reaction between free primary amines of GEL and anhydride moieties of the oligomers oPNMA or oPDMA (Figure 1). This process requires a base that buffers the protons of the carboxyl functionalities generated by the cross-linking step and does not incorporate into the material. Initially, the order of combination of these three components was investigated. In previous works, base was added as a separate component during the fabrication step and mixed with both hydrogel constituents simultaneously (direct mixing) [26,30,31,32,33]. Here, GEL and base solutions were first homogenized to a premix before addition of oligomer solution for hydrogel fabrication (GEL-base premixing). Phosphate bases were explored as a physiological alternative to the organic bases TEA or NMPO.

**Figure 2:**
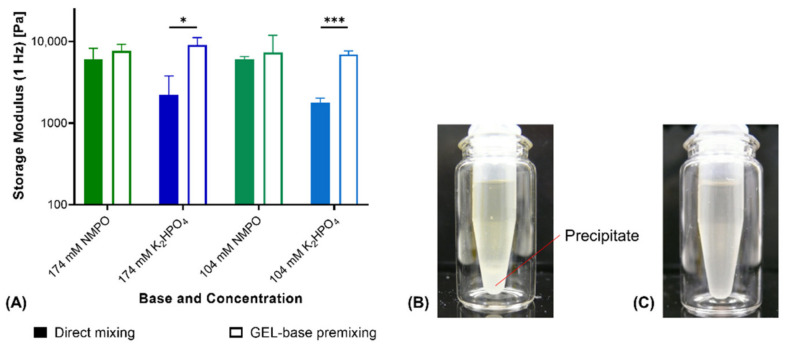
Comparison of hydrogel fabrication from separate components vs. premixing of GEL and base. (**A**) Rheological storage moduli of two-component hydrogels (15% GEL, 3.5% oPNMA-10 and either NMPO or K_2_HPO_4_ as base; *n* = 3). (**B**) Precipitate formation upon addition of base stock solution to GEL. (**C**) Dissolved precipitate after 30 s of homogenization. Results are given as mean + SD and statistical differences between groups are denoted (* represents a *p*-value less than 0.05 and *** a *p*-value less than 0.001).

With NMPO as base, fabrication with GEL-base premixing instead of direct mixing did not affect storage moduli of two-component hydrogels (Figure 2A). GEL-base premixing with potassium hydrogen phosphate (K_2_HPO_4_), however, resulted in significantly different storage moduli. Hydrogels fabricated by direct mixing were significantly less stable than gels obtained from the premix. Gels fabricated from the premix showed comparable moduli for both bases at corresponding concentration. Addition of concentrated phosphate solution to the aqueous GEL solution resulted in the formation of a white precipitate (Figure 2B). Thorough mixing for at least 30 s dissolved the precipitate and resulted in a clear, homogeneous solution (Figure 2C). Precipitation on contact of the concentrated phosphate solution with GEL suggests that the precipitate consists of a mix of phosphate and peptide. Both potassium and hydrogen phosphate ions are characterized by a high capability to induce precipitation [35]. Temporary precipitation that occurred during direct mixing with K_2_HPO_4_ led to the formation of inhomogeneous and instable gels. For that reason, GEL-base premixing was necessary for manually fabricated gels and to prepare full-gelation-mixtures for printing with K_2_HPO_4_ as base.

Next, the use of phosphates as an inorganic substitute for NMPO and TEA was investigated with oPNMA-type oligomers with 7.5, 10 and 12.5 eq of MA. In addition to K_2_HPO_4_, tripotassium phosphate (K_3_PO_4_) was also tested in an equivalent concentration (174 mM) to the other bases as well as in the halved concentration (87 mM). All hydrogels were fabricated using GEL-base premixing to ensure comparability between the tested bases and concentrations.

**Figure 3:**
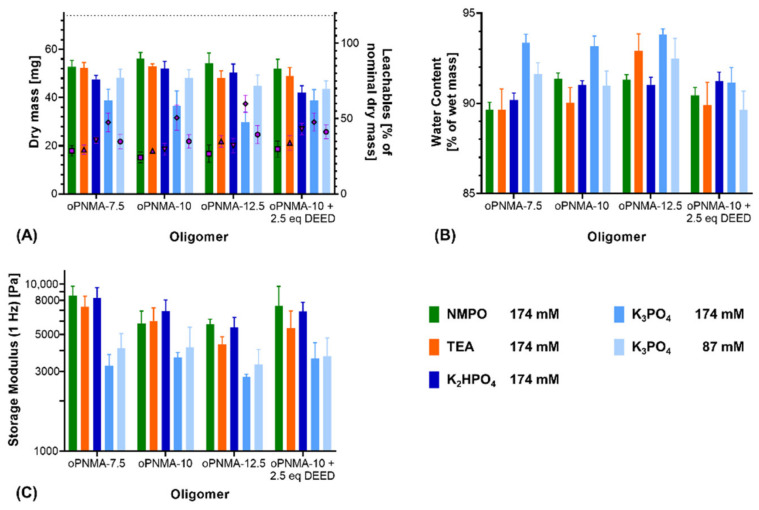
Characterization of manually fabricated two-component hydrogels with organic and inorganic bases. Two-component hydrogels were composed of 15% GEL, 3.5% of oligomer and cross-linked in presence of the evaluated base. (**A**) Salt-corrected dry masses and leachables of hydrogels after lyophilization (*n* = 5). (**B**) Water contents as fractions of the wet mass for 8 mm-disks, *n* = 5. (**C**) Storage moduli at 1 Hz for 8 mm-disks, *n* = 4–5. Results are given as mean + SD. Statistical evaluation is found in Supplementary Table S1.

Characterization of the resulting two-component hydrogels demonstrated a difference between K_2_HPO_4_ and K_3_PO_4_: For pristine oligomers, K_2_HPO_4_ in most formulations gave comparable results to NMPO and TEA regarding dry mass after lyophilization (Figure 3A) as well as water content (Figure 3B) and storage modulus of the rehydrated hydrogels (Figure 3C). Formulations with K_3_PO_4_ at both investigated concentrations (174 mM and 87 mM) resulted in gels that had significantly different characteristics (lower dry masses, higher water contents and lower storage moduli) compared to gels with either organic base or K_2_HPO_4_. Dry masses for the 87 mM K_3_PO_4_ concentration were significantly higher than for the 174 mM concentration, but still lower than when 174 mM of the organic bases were used (Figure 3A). A decreased dry mass indicated a lower incorporation of the constituent components into the hydrogel and a higher fraction of leachables extracted during the washing steps. Higher water contents in the hydrogel are likely the result of a less dense network of peptide and oligomer chains with higher amounts of interstitial water between the chains [36,37,38]. Independent of the base concentration, gels fabricated with K_3_PO_4_ were characterized by lower storage moduli than the formulation with the other three bases.

In addition to the pristine oligomers, the effect of the bases was also investigated on formulations with oPNMA-10 that had been pre-derivatized at 25% of its intact anhydrides prior to cross-linking, which is a convenient bioconjugation strategy with these oligomers [32]. Dry masses (Figure 3A), water contents (Figure 3B) and storage moduli (Figure 3C) of the resulting gels were not significantly different from either pristine oPNMA-10 or oPNMA-7.5 for the organic bases, indicating that GEL-base premixing does not significantly alter the fabrication results for the pre-derivatized oligomers [32]. Pre-derivatized oligomer with inorganic K_2_HPO_4_ resulted in a gel dry mass that was significantly lower than with pristine oPNMA-10 but was not significantly different from oPNMA-7.5 with the same base. Compared to NMPO and TEA, K_2_HPO_4_ gel dry mass was significantly reduced with either oPNMA-10 or oPNMA-7.5. No significant difference was found in gel storage moduli and water contents when comparing pre-derivatized oligomer and K_2_HPO_4_ with both pristine oligomers and organic bases. Pre-derivatizing 25% of intact anhydrides in oPNMA-10 reduces the available anhydrides for cross-linking to the level of oPNMA-7.5, which is an explanation for the lowered dry mass of pre-derivatized oPNMA-10/K_2_HPO_4_ gels compared to pristine oPNMA-10/K_2_HPO_4_. For K_3_PO_4_, dry masses and storage moduli gave similar results, with no significant difference between the pre-derivatized and pristine oligomers and significantly lower values than the organic bases. Water contents with the pre-derivatized oPNMA-10, were significantly reduced compared to the pristine oligomers and not significantly different from the organic bases. Principally, pre-derivatization of the oligomers was confirmed to work for the inorganic bases and K_2_HPO_4_ revealed to be preferable over K_3_PO_4_.

**Figure 4:**
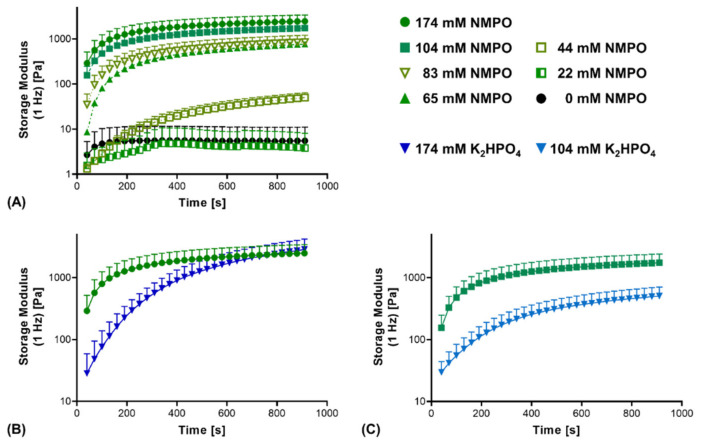
Rheological characterization of the cross-linking kinetics of the reaction between GEL and oligomer in dependence of base type and concentration. Two-component hydrogel formulations contained 15% GEL, 3.5% oPNMA-10 oligomer and different concentrations of either NMPO or K_2_HPO_4_ as base. (**A**) Cross-linking kinetics with different NMPO concentrations. (**B,C**) Kinetics of gelation in the presence of NMPO or K_2_HPO_4_ at a concentration of (**B**) 174 mM and (**C**) 104 mM. Results are given as mean + SD, with *n* = 5–15.

Additionally, the reaction kinetics between the anhydride and amine components were compared. First, reaction kinetics confirmed that insufficient base concentrations (44 mM or less) do not result in stable two-component hydrogels (Figure 4A). Comparing NMPO and K_2_HPO_4_, a significant difference in gel formation kinetics was observed. With NMPO, storage modulus increased, and hydrogel formation proceeded more rapidly than in the presence of K_2_HPO_4_. It took between 130 and 160 s for gels made with 174 mM NMPO to exceed a storage modulus of 1000 Pa, while that value was reached after approximately 430 s with the equivalent K_2_HPO_4_ concentration (Figure 4B). For a lower base concentration of 104 mM, comparable relative differences were seen between these bases (Figure 4C).

In summary, these results show that K_2_HPO_4_ was a suitable replacement for the organic bases TEA and NMPO and yielded two-component hydrogels from anhydride-containing oligomers and amine-containing gelatinous peptides with similar final properties. Interestingly, kinetics of the cross-linking reaction supported by K_2_HPO_4_ were slower than gelation processes in the presence of NMPO. This made K_2_HPO_4_ an ideal base for the two-component hydrogel mix when processability of the mix should be maintained as long as possible.

The non-physiological nature of the organic bases TEA and NMPO precluded their use for injectable, in situ gelling formulations of the two-component hydrogel [39]. In contrast, both potassium and phosphates are commonly occurring ions in living organisms and can be expected to not be cytotoxic [40,41].

### 3.2. 3D-Printing of Two-Component Hydrogel

Printing the two-component hydrogel formulations was realized by extrusion of a homogeneous mix of all three components (full-gelation-premixture) from a single cartridge. The mix was prepared immediately prior to extrusion using the GEL-base premixing protocol. Two important aspects of using the reactive full-gelation-premixture are the time necessary for the mix to become stable enough to hold its shape on the one hand and the window in which the gelling hydrogel remains printable by extrusion. For 3D-printing of the two-component hydrogels, a base concentration of 104 mM that displayed a continuously progressing gelation (Figure 4C) was chosen.

**Figure 5:**
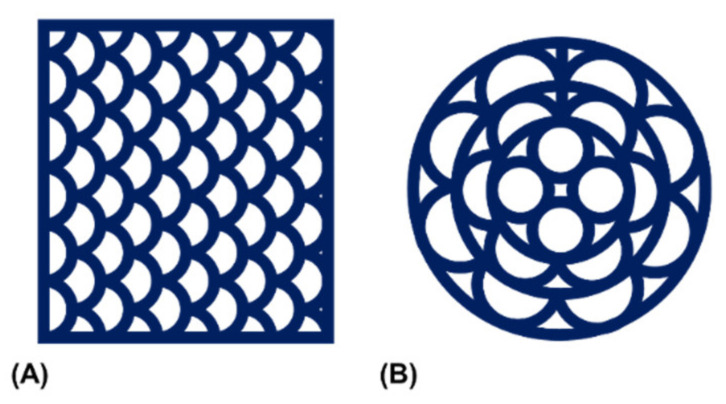
Schematic representation of the utilized printing patterns. Patterns were created to allow for continuous material extrusion with (**A**) a rectangular or (**B**) a circular peripheral outline.

The elastic behavior of the extruded hydrogel demanded a construct design that was printable without interruption. These constraints motivated fabrication of multi-channeled NGC in a transversal manner. Two printing patterns were established with a rectangular and a circular outline (Figure 5). In addition to scalability of construct height, the patterns were scalable in their horizontal dimensions and the number of printed channels.

**Figure 6:**
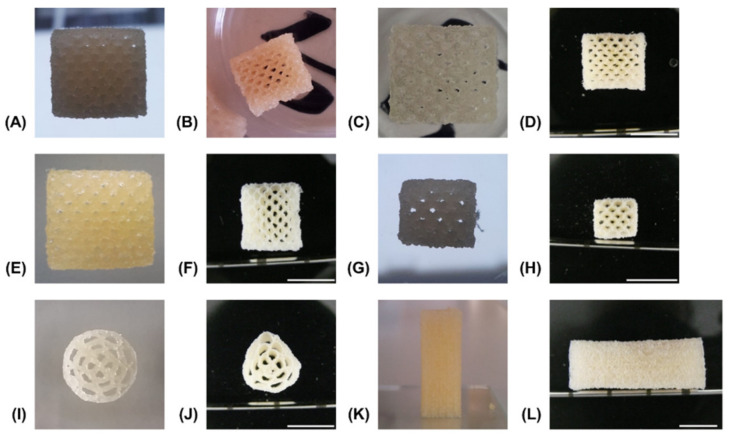
Representative pictures of generated constructs in various stages of the fabrication process. All constructs consist of two-component hydrogels composed of 15% GEL and 3.5% oPNMA-10 and were fabricated in presence of 104 mM base with a layer thickness of 0.23 mm. Visible channels in transversal view with NMPO as base, rectangular 10 × 11 mm footprint and 50 layers: (**A**) directly after printing, (**B**) after drying at normal and reduced pressure, (**C**) in wet, swollen state after washing with PBS and (**D**) dry state after lyophilization. Channels were also visible in constructs printed in the same composition and dimensions but with K_2_HPO_4_ as base: (**E**) as printed and (**F**) dry state after all processing steps. Structure of printed hydrogels of the same composition but with NMPO (dimensions: 6 × 7 mm, 30 layers): (**G**) as printed and (**H**) dry state after all processing steps. Printed hydrogels with K_2_HPO_4_ as base (dimensions: 13 mm diameter, 50 layers): (**I**) as printed and (**J**) dry state after all processing steps. Closed outer structure in lateral view of construct with 110 layers, 10 × 11 mm footprint and NMPO in (**K**) as printed and (**L**) dry state after all processing steps. Scale bar equals 10 mm.

The reference formulation composed of 15% GEL and 3.5% oPNMA-10 was used to establish a printing method and a variety of multi-channeled constructs with different dimensions were successfully printed from a single cartridge (Figure 6). Continuous channels were visible in transversal views of 50-layer constructs fabricated with NMPO directly after printing (Figure 6A), after the first drying step (Figure 6B) as well as in the equilibrium swollen hydrogel state (Figure 6C) and after lyophilization of the final hydrogel construct (Figure 6D). Open channels were seen in constructs (50-layer) fabricated with K_2_HPO_4_ directly after printing (Figure 6E) and in the freeze-dried final hydrogel construct (Figure 6F). This observation confirmed the success of printing a stable, multi-channeled structure from the two-component hydrogels and illustrated that NMPO and K_2_HPO_4_ gave similar results. In addition, structures with a smaller footprint of 6 × 7 mm were successfully generated (Figure 6G,H), as well as structures with a circular outline (Figure 6I,J). Finally, large constructs with a rectangular outline of 10 × 11 mm and 100 layers were also realized and kept their overall shape during printing (Figure 6K) and post-fabrication processing (Figure 6L). The large constructs started to indicate the limits of this printing technique. With increasing height, the mechanical strain transmitted from the moving printhead to the building platform caused an increasing motion of the construct and increasing deviation in alignment of actual needle position with the previous layer became apparent. Thus, printing resolution diminished, and the continuous structure of the smaller channels was compromised. This problem is not unique to the two-component hydrogels employed in this work, but inherent to printing flexible materials by extrusion [42]. Solutions to address this problem include an outer support structure that is manually or automatically added during the printing processor printing into a bath supporting the manufactured structure [20,43]. It is also possible to print the desired construct in smaller sections that are subsequently stacked by joining the sections in an outer conduit [44].

**Figure 7:**
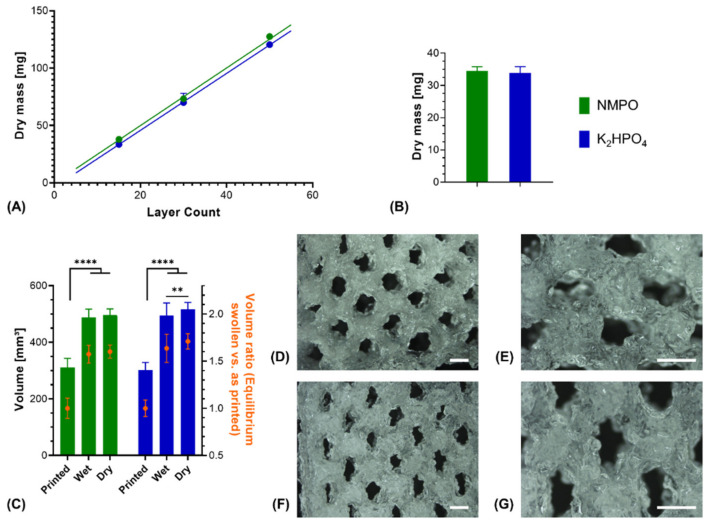
Characterization of 3D-printed two-component hydrogel constructs. All constructs consist of two-component hydrogel with 15% GEL and 3.5% oPNMA-10, with 104 mM base for fabrication, and were printed with a layer thickness of 0.23 mm. (**A**) Linear correlation of construct dry mass (10 × 11 mm rectangular outline) with number of printed layers that indicates a uniform extrusion during printing, using NMPO (*R*^2^ = 0.99) or K_2_HPO_4_ as base (*R*^2^ = 0.99) (*n* = 1–6). (**B**) Dry masses and (**C**) construct volumes (height × width × depth) of printed constructs (rectangular 6 × 7 mm footprint, 30 layers) with NMPO and K_2_HPO_4_ (absolute values and volume normalized to mean volume of freshly printed constructs, *n* = 48 for NMPO and *n* = 49 for K_2_HPO_4_). Detailed visualization of continuous open channel geometry for constructs fabricated with NMPO (**D,E**) and K_2_HPO_4_ (**F,G**). Scale bar equals 1000 µm. Results are given as mean + SD and the significance level was 95%, with ** denoting a *p*-value less than 0.01 and **** denoting a *p*-value less than 0.0001.

Printed constructs were characterized gravimetrically. Dry weights of lyophilized constructs corrected for residual salt correlated linearly with the number of printed layers in presence of either NMPO or K_2_HPO_4_ (Figure 7A). This indicated that the ongoing cross-linking reaction and resulting gelation of the two-component formulation has no discernible effect on the uniformity of material extrusion in a given time window. Consequently, it is possible to print multiple uniform constructs (rectangular, 6 × 7 mm, 30 layers) from the same mixture. No significant difference could be found in the dry masses between NMPO and K_2_HPO_4_ (Figure 7B). The swelling behavior of the two-component hydrogel constructs was macroscopically visible (Figure 6A–D) and quantified for these constructs (rectangular, 6 × 7 mm, 30 layers). Independent of the employed base, constructs had significantly higher (approximately 1.6–1.7 times) volumes in the wet, completely swollen state after washing than in the printed state (Figure 7C). The construct dimensions of the equilibrium swollen state could be maintained upon lyophilization. The additional swelling is explained by the solvent system and high GEL concentration used for printing: GEL is dissolved in water at 30% (*w/v*), while the cross-linker is dissolved in DMF. The final two-component hydrogel can bind more water than water/DMF mix (the liquid phase upon printing) and swells to its equilibrium water content of over 90% (Figure 3B) when put in a buffered aqueous medium in which DMF is extracted. Design of a multi-channel two-component hydrogel NGC would be based on the equilibrium swollen structure and the corresponding printing pattern with a swelling ratio that can be established for each combination of GEL and oligomer.

**Figure 8:**
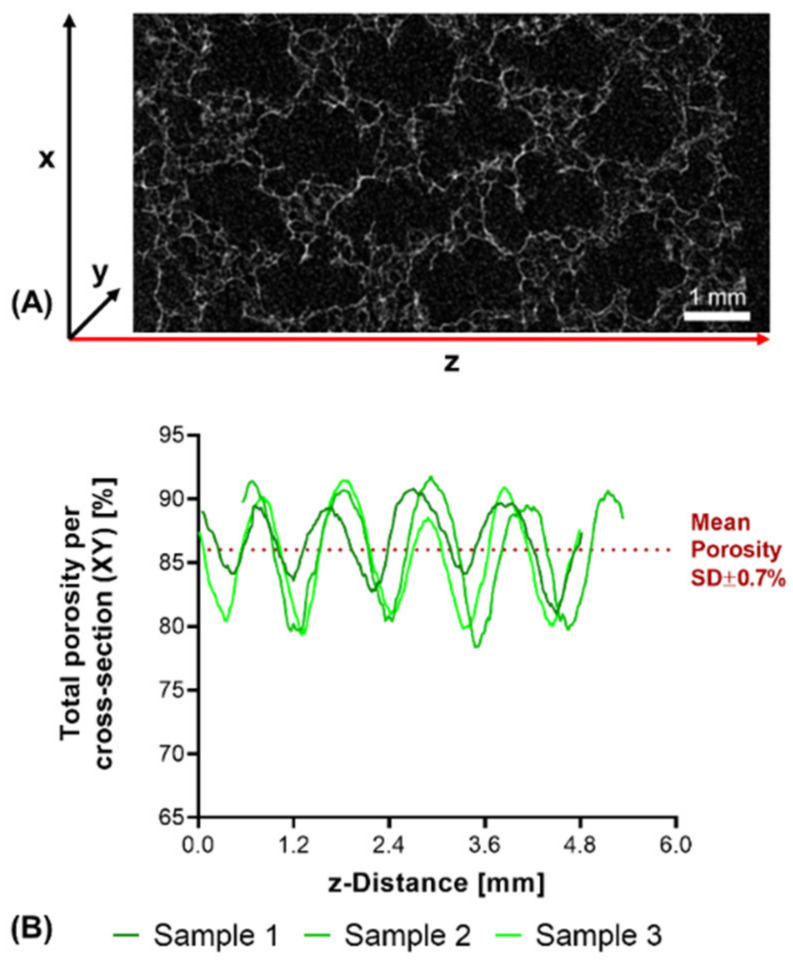
µXCT-analysis of dry, lyophilized two-component hydrogel constructs. Constructs from 15% GEL and 3.5% oPNMA-10, with 104 mM NMPO as base (dimensions: 6 × 7 mm rectangular outline, 30 layers, layer thickness: 0.23 mm). (**A**) Representative transversal cross-section, scale bar equals 1 mm. (**B**) Total porosity analysis of cross-sections (height × depth) spatially resolved along the *z*-axis (width) for three samples. Curves of sample 1 (0.03 mm) and sample 2 (0.54 mm) are offset for a better comparison of the porosity profiles. Mean porosity of all samples over the full volume is 86.0 ± 0.7%. Total porosity describes the ratio between two-component hydrogel and background voxels in every x-y-cross-section. The regularity of the overall porosity curves is directly related to the degree of order of the internal structure. SD: standard deviation.

Structural analysis of swollen constructs showed continuous open channels (Figure 7D–G). The material strands display a rather uneven and rough surface with indentations and protrusions. No discernible differences between the bases NMPO (Figure 7D,E) and K_2_HPO_4_ (Figure 7F,G) was found on these structures. With regards to later interaction with the biological environment, a rough surface is advantageous, as it provides increased surface area for cell adhesion [45]. Characterization of lyophilized constructs by µXCT displayed a structure with both macroscopic channels and smaller pores in walls that are generated by the dried two-component hydrogel strands occupying less volume than the swollen material (Figure 8A and Supplementary Video S1). The structures revealed a high overall porosity (86 ± 0.7% over the whole structure) in the dry state, with the regular structure of the channels being visible in the repetitive course of increases and decreases of construct porosity along the width of the constructs (Figure 8B). The high porosities and strand-like structures of remaining material seen for the dried two-component hydrogels are similar to observations for other hydrogel-forming materials in the dry state [46].

Taken together, these data outline the successful generation of multi-channeled structures with adaptable footprint and number of layers from a slowly gelling mix of two-component hydrogel formulations. Commercially available single-lumen NGC possess a diameter of up to 10 mm, and structures with equal or smaller sizes were realized with this printing approach [47]. This study was limited to a base concentration of 104 mM due to the motor controlling the extrusion rate not being able to extrude faster gelling compositions with higher base concentrations with the required dosing precision. Successful printing of stable structures from a slowly gelling mix demonstrates the general suitability of the two-component hydrogels for 3D-printing. Based on this proof-of-concept, future directions for development can include exploration of a more complex printhead with in-process mixing capability for use with faster gelling two-component mixes. Such a printhead would ideally possess the ability to change the ratio of the two components during printing, allowing for the generation of constructs with gradients in their composition.

### 3.3. Degradation Study with Printed Constructs

The optimal outcome of a clinical therapy with an artificial NGC would be the regeneration of nerve tissue that is functionally indistinguishable from a non-injured one. Biodegradation of the NGC in an appropriate time frame is one prerequisite to achieve this tissue quality [3]. The definition of an appropriate time frame depends on the properties of a given defect, especially defect length, diameter of the affected nerve and conditions in the surrounding tissue [1]. In this context, use of a two-component hydrogel for the NGC is interesting, as its degradation characteristics are dependent on the composition [23], and 3D-printing enables rapid generation of NGC with varied compositions. Here, general degradability of the two-component hydrogel constructs was assessed in two different degradation set-ups: The first set-up investigated degradation under purely hydrolytic conditions, while the second set-up investigated degradation in the presence of a peptide-bond cleaving enzyme.

**Figure 9:**
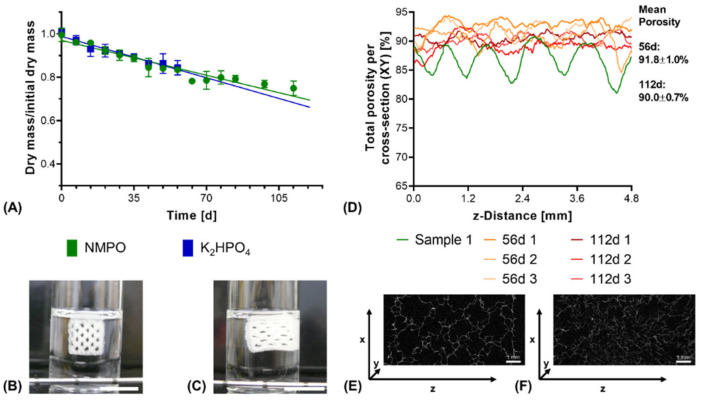
In vitro hydrolytic degradation of two-component hydrogel constructs. All constructs (6 × 7 mm rectangular outline, layer thickness 0.23 mm) consisted of two-component hydrogels composed of 15% GEL and 3.5% oPNMA-10, with 104 mM base for fabrication. (**A**) Degradation profile as dry mass relative to initial dry mass over time for non-enzymatic degradation at 37 °C (*n* = 3 per time point). Representative images for constructs in the degradation medium (**B**) at 0 days and (**C**) at 112 days. (**D**) Total porosity analysis of cross-sections of dry constructs (height x depth) spatially resolved along the *z*-axis (width) by µXCT, with a single non-degraded sample (Sample 1) for comparison. Total porosity describes the fraction of voxels in the respective x-y-cross-section not occupied by two-component hydrogel material. (**E**,**F**) Representative transversal cross-sections after 56 days (**E**) and 112 days (**F**) of degradation. Scale bar equals 1 mm.

Under exclusively hydrolytic conditions, a continuous reduction in dry mass that scaled linearly with the ongoing degradation time was observed for constructs with both bases (Figure 9A). After 56 days of degradation, both constructs with NMPO and K_2_HPO_4_ as base lost only about 15% of their initial dry mass, which is comparable to other cross-linked collagen NGC found in literature [48]. Visually, no collapse of the construct and its multi-channeled geometry could be seen even at the latest investigated time points as long as the constructs were submerged in the degradation medium (Figure 9B,C). In the dry state, µXCT imaging showed that the constructs lost the ordered, regular structure (Figure 9D) and deformed channel geometries (Figure 9E,F and Supplementary Video S2 and Supplementary Video S3) can be observed. Furthermore, degradation and structural alterations were distributed homogeneously over the entire structure, resulting in reduced mechanical integrity and a partial collapse during lyophilization.

**Figure 10:**
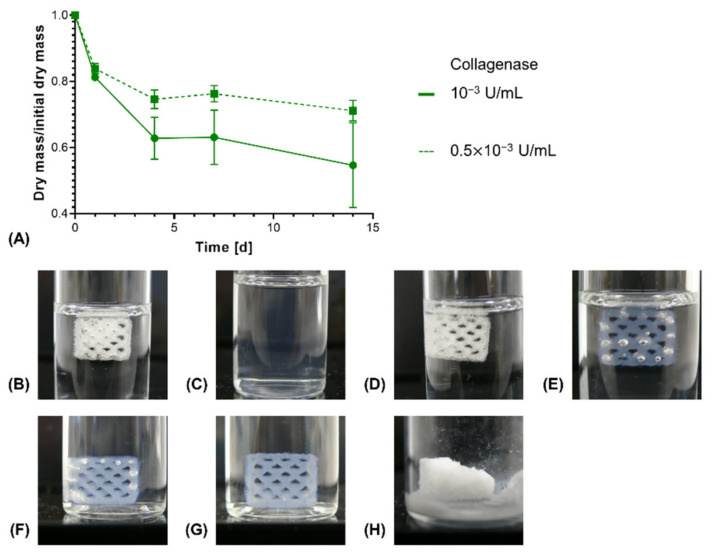
In vitro enzymatic degradation of two-component hydrogel constructs. All constructs consist of two-component hydrogels composed of 15% GEL, 3.5% oPNMA-10 and 104 mM NMPO and were printed with a 6 × 7 mm rectangular outline and a layer thickness of 0.23 mm. (**A**) Degradation profile for enzymatic degradation of constructs with NMPO as base at different initial concentrations of collagenase at 37 °C (*n* = 3 per time point). Representative images of constructs in degradation medium: (**B**) Initial state at 0 days and (**C**) after 1 day at 0.01 U/mL (data not shown in (**A**) since no dry mass could be measured after 1 day anymore); (**D**) initial state 0 days, (**E**) after 4 days, (**F**) after 7 days at 10^−3^ U/mL. (G and H) Representative images of a partially degraded construct after enzymatic degradation at 10^−3^ U/mL for 14 days (**G**) in medium and (**H**) after freeze drying.

In order to investigate enzymatic degradation, Collagenase A from *C. histolyticum* was used as a model collagen-degrading enzyme [49]. In an in vivo context, different enzymes, such as matrix metalloproteinases (MMPs), act on extracellular collagen for its remodeling [50]. Upon enzymatic degradation, an increased rate of dry mass loss was seen for constructs with NMPO as base, with the magnitude of the acceleration being dependent on the enzyme activity in the medium (Figure 10A). For 0.01 U/mL, the highest tested enzyme activity in the medium, the constructs were completely dissolved within 24 h (Figure 10B,C). This result for the anhydride-based cross-linking concept is consistent with literature data that report that high collagenase concentrations lead to complete degradation of cross-linked collagen or gelatin within a day [51,52]. For lower initial enzyme activities, no immediate collapse of channel geometry and construct structure was observed as long as the constructs maintained submerged in medium (Figure 10D–G). However, a dry mass loss of about 40–45% led to a partial collapse when the supporting medium was removed (Figure 10H). Structural collapse after a critical point of degradation is common for biomaterials displaying bulk degradation, both with synthetic and naturally derived polymers [53,54,55].

Constructs with K_2_HPO_4_ as base were also subjected to enzymatic degradation. However, base concentration was lower than the planned 104 mM due to an experimental error, which led to faster rate of degradation (Supplementary Figure S2A). Nonetheless, observations were similar to constructs with NMPO as base, with homogenous mass loss throughout the construct structure, no immediate structural collapse in the swollen state (Supplementary Figure S2B–D) and partial collapse of lyophilized structures past a dry mass loss of about 50% (Supplementary Figure S2E).

Taken together, the data indicate that the two-component hydrogels are degradable enzymatically and hydrolytically. Enzymatic degradation progressed rapidly, with a degradation rate that depended on enzyme concentration, whereas hydrolytic degradation followed a considerably slower rate. This is consistent with the published results for manually fabricated two-component hydrogel NGC, which displayed continuous hydrolytic degradation in vitro over 6 weeks but completely degraded in vivo within the same timeframe [33]. Mechanistically, hydrolytic degradation mainly occurs at the ester bonds in the oligomer structure, as the amide bonds in the primary structure of the peptides and the cross-links are less susceptible to non-catalyzed hydrolysis [56]. Conversely, collagenase and other collagen-degrading enzymes would attack the primary structure, ultimately dissolving the two-component hydrogels. In an in vivo context, the non-biodegradable remnant molecules are expected to be small enough to be excreted renally [57]. In vivo, nerve cells infiltrating the multi-channel conduit would do so primarily from the proximal ends of the defect site and remodel their immediate surrounding first [58]. The rate of degradation of an NGC should match the nerve regeneration process, in which the axonal growth cone extends at a rate of about 1 mm/day [59]. As such, the fast enzymatic degradation allows for invading cells to rapidly remodel their surrounding extracellular matrix depending on their own needs, while the slow hydrolytic degradation ensures that the two-component hydrogel is eventually completely degraded. Suitable 3D-printing techniques would also allow for the generation of NGC with gradients in hydrogel composition and adjusted degradation behavior along the length of the conduit [60]. Additionally, the two-component hydrogel can be subject to a foreign body response from the outside of the construct that leads to accelerated degradation [33], which again could be circumvented by encasing the construct in an outer conduit that is not subject to enzymatic degradation [44]. In this context, the printed two-component gel constructs can be considered as a filler for commercially available wide-lumen NGC.

### 3.4. Varying the Cross-Linking Oligomers

**Figure 11:**
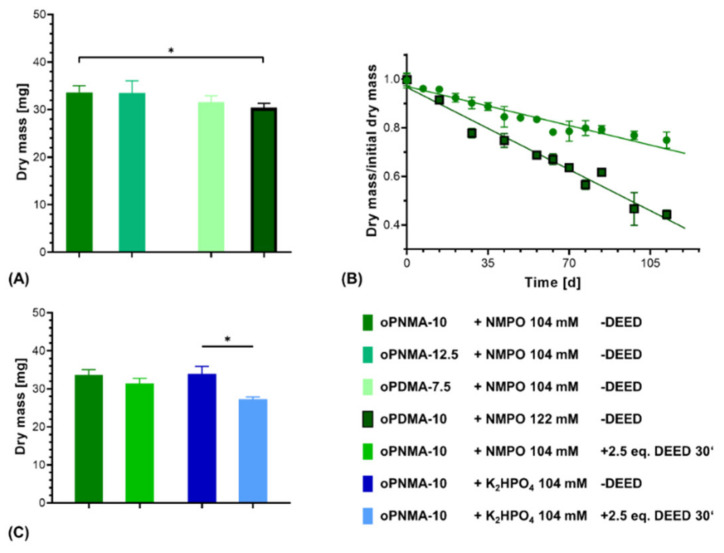
Characterization of two-component hydrogel constructs with different oligomers. All constructs consist of two-component hydrogel with 15% GEL, 3.5% oligomer and were fabricated with the indicated base concentrations and printed with a rectangular footprint and a layer thickness of 0.23 mm. (**A**) Dry masses of constructs with varying anhydride contents and co-monomers in the cross-linker (*n* = 3 for oPNMA-12.5 and oPDMA-7.5, *n* = 63 for oPNMA-10 and oPDMA-10). (**B**) Degradation profile as dry mass relative to initial dry mass over time for non-enzymatic degradation at 37 °C for oPNMA-10 (●) and oPDMA-10 (■)-consisting constructs in comparison (*n* = 3 per time point). (**C**) Dry masses of constructs with pre-derivatized cross-linker (30 min with 2.5 eq of DEED) in comparison to pristine cross-linker (*n* = 3 for derivatized samples, *n* = 63 (NMPO) and 49 (K_2_HPO_4_) for non-derivatized samples). Results are given as mean ± SD and the significance level was 95% (* denotes a *p*-value less than 0.05).

Two-component hydrogel constructs were fabricated with anhydride-containing cross-linking oligomers of different co-monomer composition (oPNMA and oPDMA) as well as with pre-derivatized oPNMA-10 in order to investigate the adaptability of the system for oligomers with different reactivities and chemical functionalities. For oPNMA-type oligomer synthesized with 12.5 eq. of MA (oPNMA-12.5), the produced constructs did not display a significant difference in dry mass loss upon degradation compared to constructs based on oPNMA-10 (Figure 11A). This is consistent with both manually fabricated gels at higher base concentrations (Figure 3) as well as previously published data for these oligomers with NMPO as the base [32] and indicates that these two cross-linking oligomers with high anhydride contents are incorporated into the constructs to a comparable degree.

With oPDMA-type oligomers, the generated constructs generally displayed a reduction in dry mass compared to the oPNMA-type oligomers (Figure 11A). For constructs with oPDMA-7.5, the dry mass difference to oPNMA-10 and -12.5 was not significant. For oPDMA-10 with the same molar anhydride-to-co-monomer ratio as oPNMA-10, no constructs could be generated upon reaction control with 104 mM NMPO. Even at a higher base concentration of 122 mM, dry masses of oPDMA-10 constructs were significantly reduced compared to the oPNMA-10-containing material. Degradation characterization showed that the oPDMA-10-containing constructs had a markedly faster loss in dry mass (Figure 11B and Supplementary Figure S3). Taken together, the lower dry mass and faster mass loss were indicative of a less well-connected hydrogel network, which can be explained by oPDMA-type oligomers containing less anhydride per mass of oligomer than oPNMA-type oligomers due to the higher molecular weight of the DAAm co-monomer [31].

Another feature of the anhydride-containing cross-linking oligomers is that a fraction of the anhydrides can be pre-derivatized with small molecules in a bioconjugation reaction immediately before their use in the hydrogel-forming reaction. Aiming to investigate the effects of pre-derivatization on formulation printability, oPNMA-10 was modified on 25% of its chemically intact anhydrides with the asymmetric diamine DEED prior to printing. Such a modification has been shown effective to improve cell adhesion to the hydrogel materials [32]. Dry mass of constructs cross-linked in the presence of NMPO was comparable with or without pre-derivatization (Figure 11C). DEED-pre-derivatized gels with K_2_HPO_4_ as base resulted in stable constructs that displayed a significantly lower dry mass than unmodified constructs. This is consistent with manually fabricated two-component hydrogels (Figure 4). However, DEED merely served as a model for an amine with which the oligomer can be pre-derivatized. Other possible amines include LM11A-31, a ligand mimicking a loop of nerve growth factor when binding to the neurotrophin 75 receptor [33], cell-interactive peptides [61] or linkers with click chemistry coupling sites for bigger molecules [62,63]. Steric hindrance of bigger pre-derivatization molecules is expected to reduce cross-linking speed and efficacy. In order to adjust construct stability for this effect, adaptations of the formulation in regard to base concentration can be tested—making use of similar effects as described for pristine oPDMA-10 above. However, it has to be considered that a change in base concentration will affect the window of printability. Therefore, such adjustments need to be balanced for both stability and printability.

The data demonstrate that premixture-based printing of the two-component hydrogels can be adapted to cross-linking oligomers with different anhydride contents, co-monomer compositions or pre-derivatization and result in stable constructs.

### 3.5. Two-Component Hydrogel Printing under Aseptic Conditions

A major requirement for implants like NGC from materials that are challenging to be sterilized in the final form, is production under aseptic conditions. A variety of regulations and guidelines exist to standardize and harmonize aseptic procedures, mainly the Good Manufacturing Practice (GMP) regulations and the methods described in the European Pharmacopeia (Ph. Eur.) [64]. In order to investigate the general feasibility of extrusion-based printing of two-component hydrogel constructs without microbiological contamination, fabrication and post-fabrication processing were performed in a laminar air flow environment with HEPA-filtered air.

Two-component hydrogel constructs were successfully fabricated under aseptic conditions. When testing for microbial contamination according to Ph. Eur. 9.0, samples are incubated in growing medium for 14 days, which is analyzed for microbial growth during and after this period. Microbial growth, indicated by medium turbidity according to Ph. Eur. 9.0, was not found in the tested formulations fabricated with either NMPO or K_2_HPO_4_ (Table 1). No difference was seen between different post-fabrication processing steps, which indicates that the washing steps and subsequent lyophilization could also be performed aseptically. This confirms that aseptic fabrication of cross-linked two-component hydrogels is generally possible, even under laboratory conditions that are not optimized for sterile production.

### 3.6. In Vitro Cell Direct Contact and Indirect Cytocompatibility Testing on Two-Component Hydrogels

For a preliminary investigation of the cytocompatibility of the two-component formulations used for printing, primary hASC were seeded and cultivated directly on manually fabricated gels. In addition, the cytocompatibility of aseptically printed constructs was assessed through indirect contact with conditioned medium, as previously published for manually fabricated gels [32].

**Figure 12:**
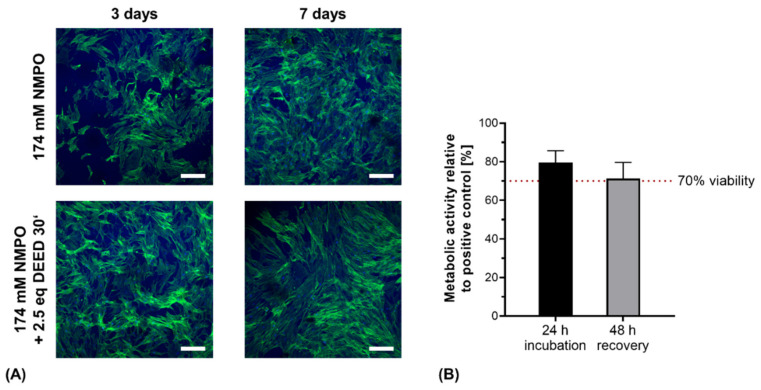
Direct contact and indirect cytocompatibility investigation of two-component hydrogels. (**A**) Primary hASC directly seeded on hydrogel discs (15% GEL, 3.5% oligomer oPNMA-10 without or with DEED pre-derivatization, cross-linked in presence of 174 mM NMPO). Laser scanning microscopic images were stained with Alexa Fluor^®^ 488 phalloidin (green, actin cytoskeleton) and DAPI (blue, nuclei). Scale bars equal 200 µm. (**B**) Indirect cytocompatibility assessment of printed two-component hydrogel constructs (15% GEL, 3.5% oPNMA-10, 104 mM NMPO). Metabolic activity was quantified as a measure of cell viability (*n* = 5). Results are given as mean + SD.

As expected from previous work, primary hASC remained vital and formed three-dimensional cell networks through the hydrogels (Figure 12A) [33]. In detail, cells revealed stretched morphologies with stress fibers in the actin cytoskeletons. These fibers indicated the presence of focal adhesion sites between cells and the gel matrix, likely mediated via integrins interacting with preserved motifs within the hydrogel. Cell proliferation and spreading appeared to be enhanced on DEED-derivatized gels though no quantification was performed at this point. This is consistent with previous results for DEED-derivatized gels with human sweat gland derived stem cells that displayed increased growth after 2 days compared to non-derivatized gels [32].

With the printed hydrogel constructs, the protocol for indirect cytocompatibility assessment was adapted for the increased mass of the printed constructs compared to hydrogel discs [32]. With aseptically fabricated, gamma-sterilized NMPO-based constructs, metabolic activity was maintained above the 70% threshold value for cytocompatibility after 24 h, proving no short-term cytotoxicity (Figure 12B). For the recovery assessment after 48 h, cell viability was not significantly reduced and remained above the critical threshold. The observed decrease in metabolic activity can be associated to less effective extraction of salt residues in the first conditioning step due to the three-dimensional, porous structure [26]. The results of direct and indirect cytocompatibility assessment with primary stem cells and fibroblasts were consistent with our previously published results for hydrogel discs [32]. More detailed biological characterization and quantification of different cell lines on the two-component hydrogels is in progress. For application of the gels in peripheral nerve regeneration, compatibility with Schwann cells that mediate the in vivo regeneration of peripheral nerve injuries will be particularly interesting [65].

## 4. Conclusions

This work demonstrated, for the first time, the ability to successfully employ oligomer-cross-linked two-component hydrogels from gelatinous peptides for the generation of multi-channeled structures by extrusion-based 3D-printing. Stable constructs were printed with cross-linking oligomers with different anhydride contents, co-monomer compositions as well as partial pre-derivatization, indicating a robust adaptability of the printing strategy. Furthermore, it was demonstrated that the biocompatible inorganic base K_2_HPO_4_ is an effective substitute for the organic bases TEA and NMPO in the production of two-component hydrogels in techniques for both manual fabrication and extrusion-based printing when the solid base was thoroughly mixed with the gelatinous peptide solution prior to addition of the oligomer component. The hydrogel constructs were shown to be degradable by both hydrolytic and enzymatic approaches in vitro, and aseptic fabrication under laboratory conditions was possible. Taken together, the work outlined both the potential of the material for 3D-printing as well as future challenges in regard to material composition and printing set-up.

## Figures and Tables

**Table 1:** Test for microbiological contamination of aseptically fabricated two-component hydrogel constructs according to Ph. Eur. 9.0, monograph 2.6.1.

NMPO		K_2_HPO_4_	
Post-Fabrication	Result	Post-Fabrication	Result
Standard protocol (SP)	No growth (0/6 samples)	Standard protocol (SP)	No growth (0/6 samples)
SP w/o washing and lyophilization	No growth (0/3 samples)	SP w/o washing and lyophilization	No growth (0/3 samples)
SP plus γ-sterilization	No growth (0/6 samples)	SP plus γ-sterilization	No growth (0/6 samples)

All constructs consist of two-component hydrogel with 15% GEL, 3.5% oPNMA-10 and were fabricated in presence of 104 mM of the indicated base with a rectangular 6 × 7 mm footprint and 30 layers with a thickness of 0.23 mm. Samples were fabricated following the standard protocol and variations thereof: standard protocol (printing of construct, drying under reduced pressure, washing with sterile PBS and lyophilization), standard protocol w/o washing and lyophilization, standard process and subsequent γ-sterilization with 15 kGy.

## Data Availability

Data available on request.

## Supplementary Materials

The following are available online at https://www.mdpi.com/article/10.3390/biomedicines9040370/s1: Methods and results for characterization of the cross-linking oligomer in detail (Supplementary Table S1), statistical evaluation of the characterization of manually fabricated two-component hydrogels (Supplementary Table S2), structural elements and synthesis scheme of anhydride-containing oligomers (Supplementary Figure S1), enzymatic degradation of constructs with K_2_HPO_4_ as base (Supplementary Figure S2) and oPDMA-10 cross-linked two-component hydrogel constructs (Supplementary Figure S3), 3D-reconstruction of µXCT data for nondegraded (Supplementary Video S1), 56 d (Supplementary Video S2) and 112 d (Supplementary Video S3) hydrolytically degraded constructs.
