# Supplementary material for: Extrusion-Printing of Multi-Channeled Two-Component Hydrogel Constructs from Gelatinous Peptides and Anhydride-Containing Oligomers
Source: Biomedicines. 2021 Apr 1;9(4):370. doi: 10.3390/biomedicines9040370 (PMC8065526; doi:10.3390/biomedicines9040370)
Supplement: Supplementary file 1 [file biomedicines-09-00370-s001.zip › biomedicines-1142640-supplementary.docx]

*Electronic Supplementary Information*

Extrusion-printing of multi-channeled two-component hydrogel constructs from gelatinous peptides and anhydride-containing oligomers

Jan Krieghoff^a^, Johannes Rost^b^, Caroline Kohn-Polster^a^, Benno M. Müller^a^, Andreas Koenig^c^, Tobias Flath^b^, Michaela Schulz-Siegmund^a^, Fritz-Peter Schulze^b^, Michael C. Hacker^a,d^*

Affiliation:

^a^ Institute of Pharmacy, Pharmaceutical Technology, Faculty of Medicine, University of Leipzig, Eilenburger Straße 15a, 04317 Leipzig, Germany

^b^ Department of Mechanical and Energy Engineering, Leipzig University of Applied Sciences (HTWK Leipzig), Karl-Liebknecht-Straße 134, 04277 Leipzig, Germany

^c^ Department of Prosthodontics and Materials Science, University of Leipzig, Liebigstraße 12, 04103 Leipzig, Germany

^d^ Institute of Pharmaceutics and Biopharmaceutics, Heinrich Heine University, Universitätsstraße 1, 40225 Düsseldorf, Germany

*Corresponding Author:

Michael C. Hacker

Heinrich Heine University

Institute of Pharmaceutics and Biopharmaceutics

Universitätsstraße 1

40225 Düsseldorf, Germany

Phone: +49 211 81-14220

mch@mchlab.de**Table of Contents:**

Total pages: 9

Total Tables: 2

Total Figures: 3

Total Videos: 3

**Materials and Suppliers**  S3

**Methods for characterization of cross-linking oligomers**  S3

**Structural elements of anhydride-containing cross-linkers** S5

**Results for characterization of cross-linking oligomers**  S6

**Supplementary Figures and Tables**  S7

**References** S10

Materials and Suppliers

Pentaerithrytol diacrylate monostearate (PEDAS), *N*-isopropylacrylamide (NiPAAm), azobisisobutyronitrile (AIBN), elemental potassium, triethylamine (TEA), *N*-methylpiperidin-3-ol (NMPO), Dulbecco’s Modified Eagle Medium (DMEM) with low and high glucose, 200 mM alanyl glutamine solution, Triton X-100, paraformaldehyde, bovine serum albumin and goat serum were purchased from Sigma-Aldrich Germany. Maleic anhydride (MA), benzophenone, aniline, potassium hydrogen phosphate and tripotassium phosphate were purchased from Acros Organics. Diacetone acrylamide (DAAm) was purchased from Tokyo Chemical Industry. Tetrahydrofuran (THF), dichloromethane (DCM) and acetone were purchased from VWR. Elemental sodium, potassium hydrogen phthalate, benzoic acid, sodium azide and forskolin were acquired from Merck KGaA (Darmstadt, Germany). Diethyl ether was purchased from Carl Roth GmbH. CDCl_3_ and d6-dimethyl sulfoxide (DMSO) containing 0.03 % of tetramethylsilane (TMS) were acquired from Armar Europe GmbH. Ethanol (96% (V/V)), *N,N*-dimethylformamide (DMF) and phosphate-buffered saline (PBS) powder were purchased from PanReac AppliChem. Gelatinous peptides from partially hydrolyzed collagen (Collagel^®^, GEL, type B, Lot #895050V, average molecular weight 12.6 kDa) were kindly provided by Gelita AG (Eberbach, Germany). Collagenase A from *C. histolyticum* was purchased from Roche AG (Switzerland). Precision silicon O-rings were acquired from RS Components GmbH (Mörfelden-Walldorf, Germany) and HUG^®^ Technik und Sicherheit GmbH (Ergolding, Germany). Fetal bovine serum, penicillin/streptomycin were purchased from PAA Laboratories (Pasching, Austria). DAPI and AlexaFluor^®^ 488 phalloidin were bought from Invitrogen (Darmstadt, Germany). L929 fibroblasts were acquired from Cell Lines Service (Eppelheim, Germany). Human adipose tissue-derived stromal cells (hASCs) were obtained from liposuction aspirates of healthy donors with written consent and ethical committee approval as published previously [1].

Methods for Characterization of Anhydride-Containing Oligomers (Cross-Linker)

1. Proton Nuclear Magnetic Resonance (^1^H-NMR)

Nuclear magnetic resonance spectroscopy was performed on a Varian MERCURYplus 300 spectrometer. Oligomer samples were dissolved to 20 mg/mL in a 2:1 (v/v) mixture of CDCl_3_ and d6-DMSO, with 0.03% TMS serving as an internal reference. Processing of acquired data was performed with the MestReNova software package (version 11, Mestrelab Research S.L., Spain).

2. Gel Permation Chromatography (GPC)

The molecular weight distribution of the synthesized oligomers was analyzed using a polymer-based analytical column combination (SDV combination medium, 5 µm particle size, 8x50 mm guard column, 8x300 mm 1000 Å column, 8x300 mm 100’000 Å column, PSS Polymer Standards Service GmbH, Germany) in a SECcurity GPC system (Agilent Technologies, Boeblingen, Germany) with THF as the solvent. The GPC system contained a degasser, a pump, an autosampler/injector, a column oven and detectors for molecular weight, refractive index and viscosity and was controlled by the WinGPC Unity software (build 6807, PSS). Samples were dissolved in THF (10 mg/mL) and filtered (0.45 µm, PTFE, VWR) prior to analysis. Oligomer molecular weights were determined relative to a polystyrene standard kit (ReadyCal Kit, PSS). With the Unity software, number average (Mn) and weight average (Mw) molecular weights and dispersity (Ð_M_ = Mw/Mn) were calculated for each oligomer.

3. Maleic Anhydride Content and Intactness

The incorporation of maleic anhydride into the oligomers and the chemical intactness of the anhydrides were investigated by conductometric and Brown-Fujimori titrations.

3.1. Conductometric Titration

The overall incorporation of MA was calculated using acidimetric titration to determine the number of carboxy functionalities following complete anhydride hydrolysis. Weighed oligomer samples were dissolved in 50 mL acetone. Following addition of 50 mL DI water, samples were covered and stirred at 60°C for 2 h to completely hydrolyze all anhydride moieties. After temperature adjustment, samples were titrated with 0.1 M aqueous NaOH solution (standardized with potassium hydrogen phthalate) at constant temperature, while conductivity was monitored (CO 3000 H conductivity meter, VWR). Each oligomer was measured in triplicate. The overall content of anhydride moieties in the oligomer is equivalent to half the number of carboxyl functionalities.

3.2. Brown-Fujimori Titration

A titration scheme derived from the protocol developed by Brown and Fujimori [2] was employed for determination of the amount of intact anhydrides. Weighed oligomer samples were dissolved in 10 mL freshly distilled THF prior to addition of 5 mL aniline. After stirring for 5 min, 50 mL 96% ethanol were added to the solution. The samples were then titrated with a 0.05 M solution of NaOH in 96% ethanol (standardized with benzoic acid). The result at equivalence point is the number of carboxyl functionalities in the oligomer if all intact anhydrides are derivatized with an excess of aniline.

3.3. Anhydride Intactness

With the overall content of maleic anhydride ([MA]_CT_) and the number of carboxyl functionalities after derivatization with aniline ([COOH]_BFT_), the fraction of intact anhydrides in the cross-linking oligomers can be calculated. As derivatization of an anhydride moiety results in one carboxyl functionality while hydrolysis of an anhydride moiety yields two carboxyl functionalities, the fraction of intact anhydrides can be calculated according to the following equation (1).

| 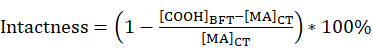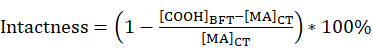 | (1) |
| --- | --- |

Structural elements of anhydride-containing cross-linkers


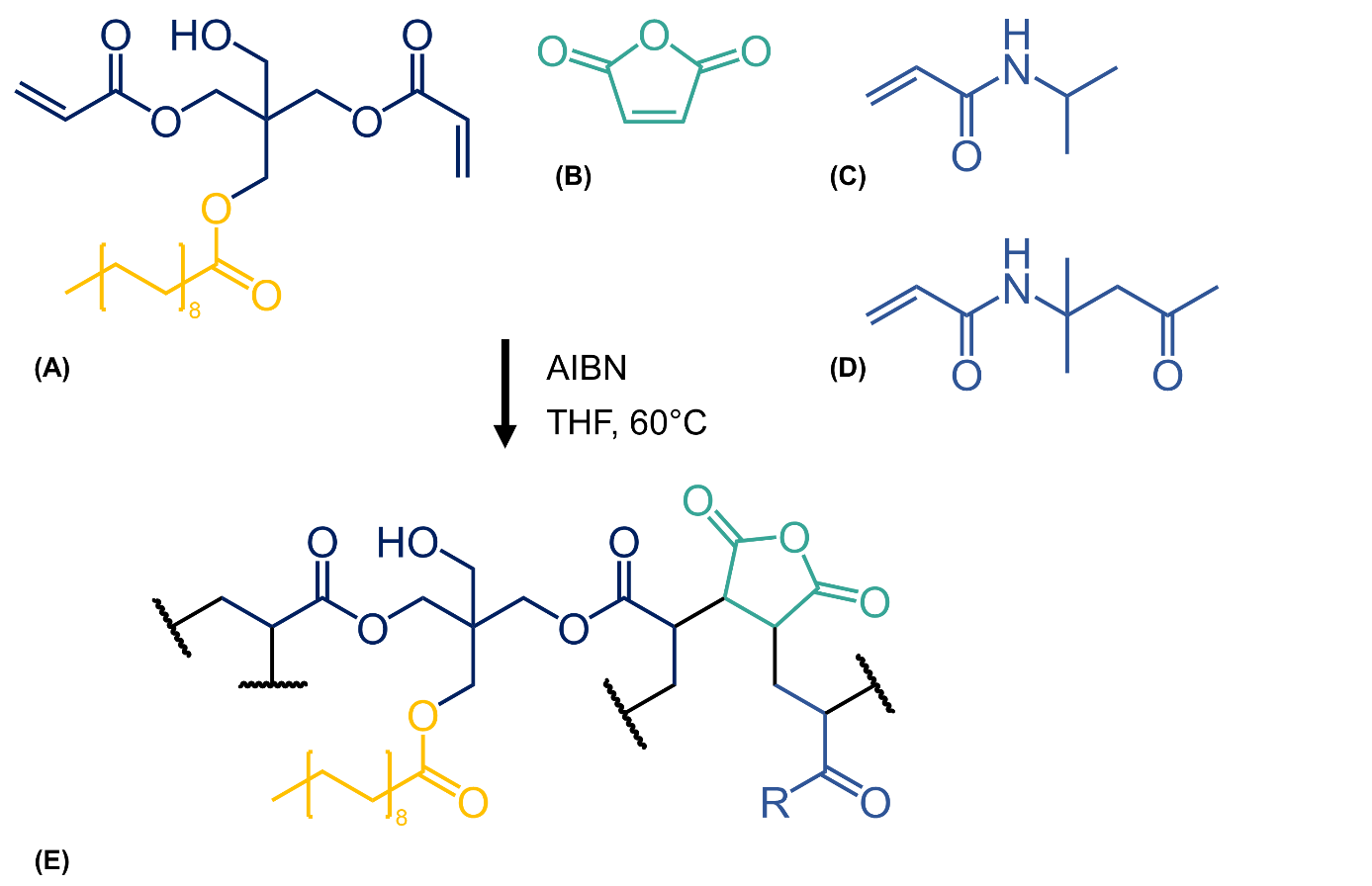


Supplementary Figure S1: Synthesis scheme for anhydride-containing oligomers. (**A–D**) Educts with unsaturated double bonds: (**A**) Pentaerythritol diacrylate monostearate (PEDAS); (**B**) maleic anhydride (MA); (**C**) *N*-isopropylacrylamide (NiPAAm); (**D**) diacetone acrylamide (DAAm). (**E**) Oligomer oPNMA (oligo(PEDAS-*co*-NiPAAm-*co*-MA)) (synthesized from A, C and B) or oPDMA (oligo(PEDAS-*co*-DAAm-*co*-MA)) (synthesized from A, D and B). AIBN: azobisisobutyronitrile (catalyst); THF: tetrahydrofuran (solvent).

Results for Characterization of Cross-Linking Oligomers

The oPNMA- and oPDMA-type oligomers synthesized with an MA feed of 7.5, 10 and 12.5 molar equivalents (oPNMA-7.5, -10, -12.5 and oPDMA-7.5 and -10) were polymerized as previously described [3,4]. All synthesized cross-linking oligomers were characterized for monomer incorporation, molecular weights and anhydride intactness by proton NMR, GPC and conductometric titration, respectively (Suppl. Tab. 1). Oligomer anhydride incorporation scaled with the reaction feed of MA. Anhydride intactness was found to range between 75%–85% for oPNMA-type oligomers and around 70% for oPDMA-type oligomers. Both number- and weight-average molecular weights decreased with increasing MA feed, while dispersity (ratio of weight-average to number-average molecular weight) was found unaffected by oligomer type and composition. All of these characteristics have been expected from previous work with these oligomers [3,4].

**Supplementary Table 1.** Composition and molecular weight characterization of synthesized oPNMA- and oPDMA-oligomers. Oligomer composition by (^a^) ^1^H-NMR and (^b^) conductometric/Brown-Fujimori titrations as well as molecular weight by GPC. Ð_M_: molecular weight dispersity, calculated as the ratio of M_w_ to M_n_.

| **Type** | **Oligomer Composition** | | | |  | **Molecular Weight (GPC)** | | |
| --- | --- | --- | --- | --- | --- | --- | --- | --- |
|  | **NiPAAm/ DAAm content (relative to PEDAS)^a^** | **MA content^b^** | | |  |  |  |  |
|  |  | **[mmol/g oligomer]** | **[wt-%]** | **Intact MA [%]** |  | **M_n_ [Da]** | **M_w_ [Da]** | **Ð_M_** |
| oPNMA-7.5 | 10.7 | 2.13 ± 0.10 | 20.9 ± 1.0% | 78.0% |  | 2290 ± 150 | 3533 ± 74 | 1.5 ± 0.1 |
| oPNMA-10 | 11.8 | 2.49 ± 0.07 | 24.4±0.7% | 80.4% |  | 1721 ± 93 | 2784 ± 125 | 1.6 ± 0.1 |
| oPNMA-12.5 | 9.9 | 2.82 ± 0.03 | 27.7±0.3% | 83.0% |  | 1424 ± 94 | 2143 ± 90 | 1.5 ± 0.0 |
| oPDMA-7.5 | 19.2 | 1.70 ± 0.01 | 16.6 ± 0.1% | 66.1% |  | 2319 ± 18 | 3954 ± 40 | 1.7 ± 0.0 |
| oPDMA-10 | 18.4 | 2.06 ± 0.02 | 20.2 ± 0.1% | 70.1% |  | 1665 ± 31 | 2713 ± 56 | 1.6 ± 0.0 |

Supplementary Figures and Tables

**Supplementary Table 2.** Statistical evaluation of the characterization of manually fabricated two-component hydrogels with organic and inorganic bases (Figure 2). Hydrogels were fabricated from 15% GEL, 3.5% of the respective cross-linking oligomer and the respective base. Pre-derivatization with DEED was performed for 30 min. Salt-corrected dry masses and leachables of complete hydrogels after lyophilization, *n* = 5; water contents as fractions of the wet mass for 8 mm-disks, *n* = 5; rheological storage modulus at 1 Hz for undamaged 8 mm-disks, *n* = 4–5. Pairwise evaluation was performed by Tukey’s post-hoc test following two-way ANOVA in Graphpad Prism 8 at a significance level of 0.05. *: *p* < 0.05; **: *p* < 0.01; ***: *p* < 0.001; ****: *p* < 0.0001.


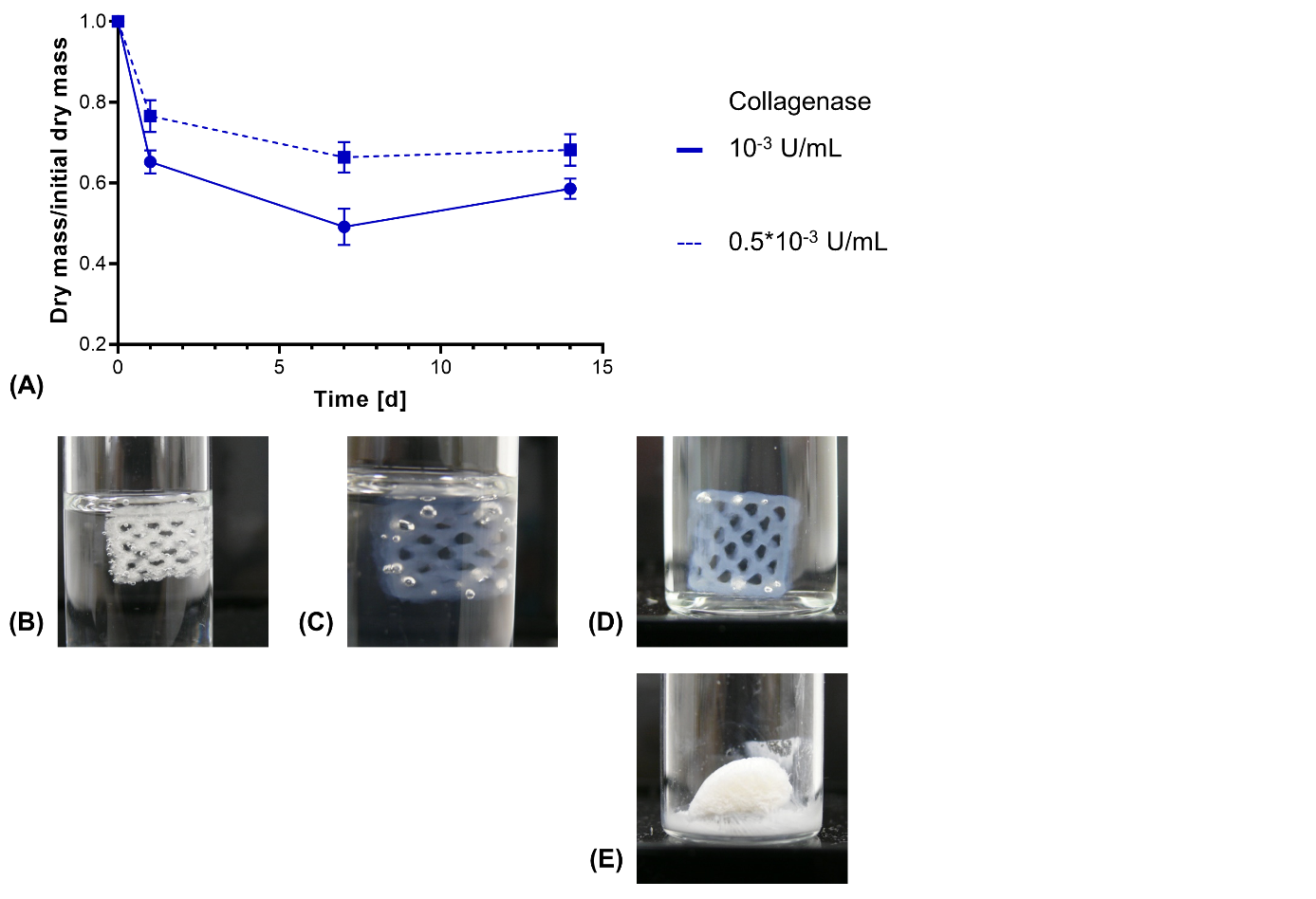


**Supplementary Figure S2.** In vitro enzymatic degradation of two-component hydrogel constructs with 15% GEL, 3.5% oPNMA-10 and 104 mM K_2_HPO_4_, printed with a 6 × 7 mm rectangular footprint and 30 layers a 0.23 mm thickness. (**A**) Degradation profile for enzymatic degradation of constructs at different initial concentrations of collagenase at 37°C. Representative images of constructs in degradation medium: (**B**) initial state (day 0), (**C**) after 7 days and (**D**) after 14 days at 10^-3^ U/mL. (**E**) Representative image of partially degraded construct after enzymatic degradation at 10^-3^ U/mL for 14 days in the lyophilized, partially collapsed state.


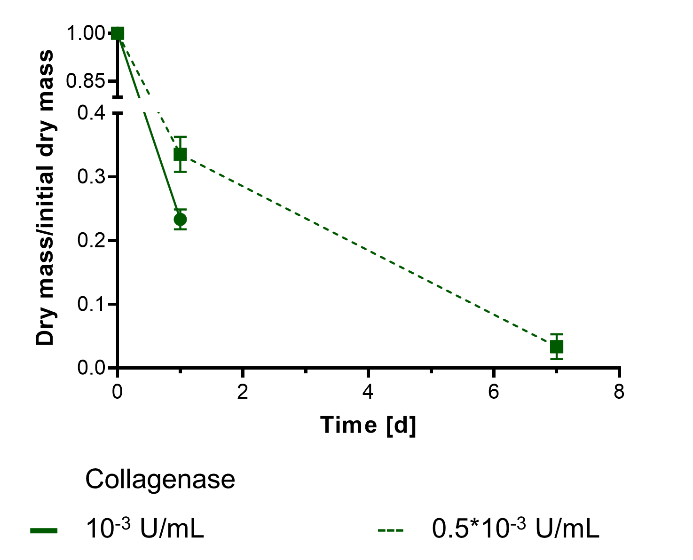


**Supplementary Figure S3.** In vitro enzymatic degradation of two-component hydrogel constructs with 15% GEL and 3.5% oPDMA-10, fabricated in the presence of 122 mM NMPO, printed with a 6 × 7 mm rectangular footprint and 30 layers with 0.23 mm thickness. Degradation profile for enzymatic degradation of constructs at different initial concentrations of collagenase at 37 °C.

Supplementary Videos - Descriptions

Supplementary Video S1: Rotation of the three-dimensional µXCT data reconstruction of an exemplary dry, lyophilized construct (15% GEL, 3.5% oPDMA-10, 104 mM NMPO, printed with a 6x7 mm rectangular footprint and 30 layers with 0.23 mm thickness). Open channels are visible in the lateral view.

Supplementary Video S2: Rotation of the three-dimensional µXCT data reconstruction of an exemplary dry, lyophilized construct after 56 days of degradation (15% GEL, 3.5% oPDMA‑10, 104 mM NMPO, printed with a 6x7 mm rectangular footprint and 30 layers with 0.23 mm thickness). Partial collapse of the dried constructs is visible, with only few channels being open all the way through the construct.

Supplementary Video S3: Rotation of the three-dimensional µXCT data reconstruction of an exemplary dry, lyophilized construct after 112 days of degradation (15% GEL, 3.5% oPDMA‑10, 104 mM NMPO, printed with a 6x7 mm rectangular footprint and 30 layers with 0.23 mm thickness). No open channels are visible in the reconstruction, indicating partial collapse of the construct when the supporting medium is removed.

References

1. H.; Schneider, B.; Sedaghati, A.; Naumann, M.C.; Hacker, M. Schulz-Siegmund, Gene silencing of chordin improves BMP-2 effects on osteogenic differentiation of human adipose tissue-derived stromal cells, Tissue Eng. Part A 20 (2014) 335–345. https://doi.org/10.1089/ten.TEA.2012.0563.
2. A.; Brown, K.; Fujimori, A method for the determination of maleic anhydride content in copolymers, Polymer Bulletin 16 (1986) 441–444. https://doi.org/10.1007/BF00955576.
3. T.; Loth, R.; Hennig, C.; Kascholke, R.; Hötzel, M.C.; Hacker, Reactive and stimuli-responsive maleic anhydride containing macromers – multi-functional cross-linkers and building blocks for hydrogel fabrication, Reactive and Functional Polymers 73 (2013) 1480–1492. https://doi.org/10.1016/j.reactfunctpolym.2013.08.002.
4. C.; Kascholke, T.; Loth, C. Kohn-Polster, S.; Moller, P.; Bellstedt, M. Schulz-Siegmund, M.; Schnabelrauch, M.C.; Hacker, Dual-Functional Hydrazide-Reactive and Anhydride-Containing Oligomeric Hydrogel Building Blocks, Biomacromolecules 18 (2017) 683–694. https://doi.org/10.1021/acs.biomac.6b01355.
